# Evolutionarily Conserved Sequence Features Regulate the Formation of the FG Network at the Center of the Nuclear Pore Complex

**DOI:** 10.1038/srep15795

**Published:** 2015-11-06

**Authors:** M. Peyro, M. Soheilypour, B.L. Lee, M.R.K. Mofrad

**Affiliations:** 1 Molecular Cell Biomechanics Laboratory, Departments of Bioengineering and Mechanical Engineering, University of California, Berkeley, CA 94720.

## Abstract

The nuclear pore complex (NPC) is the portal for bidirectional transportation of cargos between the nucleus and the cytoplasm. While most of the structural elements of the NPC, i.e. nucleoporins (Nups), are well characterized, the exact transport mechanism is still under much debate. Many of the functional Nups are rich in phenylalanine-glycine (FG) repeats and are believed to play the key role in nucleocytoplasmic transport. We present a bioinformatics study conducted on more than a thousand FG Nups across 252 species. Our results reveal the regulatory role of polar residues and specific sequences of charged residues, named ‘like charge regions’ (LCRs), in the formation of the FG network at the center of the NPC. Positively charged LCRs prepare the environment for negatively charged cargo complexes and regulate the size of the FG network. The low number density of charged residues in these regions prevents FG domains from forming a relaxed coil structure. Our results highlight the significant role of polar interactions in FG network formation at the center of the NPC and demonstrate that the specific localization of LCRs, FG motifs, charged, and polar residues regulate the formation of the FG network at the center of the NPC.

The nuclear pore complex (NPC) is the sole gateway for bidirectional transport of cargo between the cytoplasm and nucleus^1^. Fast, yet highly selective, translocation of vital cargos ranging from different functional proteins to RNAs and ribosomes is the most interesting and distinctive property of the nuclear pore supramolecular machine, which has attracted extensive attention over the past few decades^1^,^2^. Each NPC can handle up to 1000 translocations per second via two modes of nucleocytoplasmic transport, namely active and passive^1^. While passive transport is simply free diffusion of smaller cargos through the NPC, active transport requires a family of transporters, i.e. karyopherins (Kaps), to attach to cargos and carry them across the pore. It is suggested that whether a molecule is transported passively or actively depends on its size, with small molecules (molecular mass M ~ 20–40 kDa, diameter ~5–9 nm) passively diffusing through the pore while larger molecules (M > 40 kDa up to ~25 MDa, diameter of up to ~40 nm) need transporters^1^,^3^,^4^.

Nucleoporins (Nups) are the building blocks of the nuclear pore, with each NPC consisting of approximately 30 different types of Nups^5^. Approximately one-third of these proteins are rich in phenylalanine-glycine repeats, hence termed FG Nups, and possess highly flexible structures^1^,^6^. FG Nups are categorized as intrinsically disordered proteins (IDPs), i.e. they comprise domains that exhibit little or no secondary structure^5^,^6^,^7^. FG repeats constitute more than 29% of sequences of FG Nups^8^, indicating the substantial role of these regions in transport. Weak and transient hydrophobic interactions between transporters and FG motifs, embedded in disordered domains of FG Nups, are the main driving forces that stimulate active translocation of cargos through the NPC^9^.

Due to the complex and highly dynamic nature of the NPC and its constituents, current imaging and experimental techniques are unable to fully capture a detailed picture of the function and mechanics of FG Nups^10^,^11^. Computational methods can be effective in predicting the *in vitro* and *in vivo* behavior and function of FG Nups in different spatiotemporal scales. There is a growing body of computational studies looking through the nuclear pore via different perspectives. Some studies have specifically focused on modeling of the behavior of single FG Nups. Krishnan *et al.* studied the FG domains of Nup116 using molecular dynamics (MD) simulations^12^. Devos *et al.* combined computational and biochemical methods to assign folds to some 30 different types of proteins found in the yeast NPC^8^. Other studies examined the interaction between Nups and Kaps. For example, Zhao *et al.* investigated the interaction of CRM1 with Tpr, a nuclear pore protein located inside the nuclear basket, and identified nine possible binding sites for Tpr on CRM1^13^. Aggregation and interaction of FG Nups has been the focus of other computational studies. For example, Dolker *et al.* conducted MD simulations on a few FG repeat sequences and investigated the aggregation of FG Nups and the role of hydrophilic linkers in this phenomenon^14^. Miao and Schulten performed MD analysis on a group of FG Nups grafted on a planar surface^3^. Using a computational framework, Alber *et al.* determined the molecular architecture of NPC from a set of biophysical and proteomic data^15^,^16^. Wolf *et al.* performed an analytical/computational analysis on the significance of eight-fold symmetry of the NPC scaffold^17^. Using a coarse-grained model, Ando *et al.*^18^ performed a comprehensive study on the effect of the sequence on the morphology of single FG Nups and grafted rings of FG Nups. In addition, there are several studies focused on the modeling of the NPC and its constituents as a whole. Ghavami *et al.* investigated the distribution of FG Nups in the channel by means of a high resolution coarse-grained molecular dynamics model and showed that the distribution is encoded in the sequences of FG Nups^19^. Tagliazucchi *et al.*^20^ also used a computational model to simulate the effect of sequences of FG Nups on translocation through the NPC. Other studies incorporated the transporters in addition to the NPC and its constituents, in an effort to provide a more comprehensive understanding of the NPC. Mincer and Simon used the pairwise agent interaction with rational superpositions (PAIRS) model to predict factors such as the bimodal cargo distribution, the effect of the number of NLS tags and RanGTP gradient^21^. They modeled rings of Nups and the NPC as a whole and verified their model using a variety of experimental data. Osmanovic *et al.* modeled FG Nups as freely jointed chain polymers to explore the effect of NTRs on polymeric structure in the NPC^22^. Moussavi-Baygi *et al.* studied the functional state of the NPC and measured transportation time versus cargo size via a coarse-grained Brownian dynamics model^11^,^23^. Zilman *et al.* developed a mathematical model of the NPC to discuss robustness, efficiency and selectivity of the NPC^24^. Colwell *et al.*^25^ investigated the electrostatic interactions between FG Nups and transporters and found that these interactions play a key role in nucleocytoplasmic transport, as transporters contain negative charge and the NPC’s permeability barrier is positively charged. Patel *et al.*^26^ investigated the role of different FG domains in forming the permeability barrier in yeast FG Nups. Using agent-based modeling (ABM), Azimi at al. argued that the presence of an affinity gradient between Kap and FG Nups across the NPC maximizes the transport rate through the nuclear pore^27^. Azimi *et al.* further extended their ABM model to study the dynamics of mRNA export through the nuclear pore^28^. Although these studies shed light on the behavior of specific domains of FG Nups, further examinations are needed to capture a comprehensive picture of the overall characteristics, behavior and function of Nups inside the NPC.

Arguably, the well-known correlation between sequence, structure and function in proteins does not hold in the case of IDPs^29^,^30^,^31^,^32^,^33^,^34^,^35^,^36^. As a result, many studies have incorporated non-classical approaches, e.g. sequence composition and physical property distribution along the sequence, to decipher behavior and function of IDPs^37^,^38^,^39^. Therefore, the classical correlation between sequence, structure and function for structured proteins would be translated to a relationship between sequence composition, structural properties and function in IDPs^39^,^40^. Ando *et al.*^39^ performed a bioinformatics study on a database of 1138 FG Nups from 252 species and found important evolutionarily conserved features in FG Nups regarding FG motifs and charged clusters density and location. A slightly modified version of this database was adopted in the present study for a deeper analysis of the distribution of different types of residues along the sequences of FG Nups. We analyzed the number density (the number of a specific type of residue divided by the length of the entire sequence) and the location of polar residues and discovered specific sequences of charged residues common in all FG Nups.

In this study, multiple bioinformatics approaches are adopted with the aim to uncover unique features in the sequences of FG Nups. A large database of FG Nups is used to extract their evolutionarily conserved characteristics. Comparing FG Nups against a database of experimentally approved disordered proteins, i.e. DisProt^41^, it is observed that there is a considerable distinction in number density and distribution of charged and polar residues among the two groups of proteins. A more in-depth analysis of FG Nup sequences reveals that being rich in FG repeats is not the only evolutionarily conserved property the FG Nups possess. Our sequence analysis demonstrates a specific distribution of charged residues in the sequences of FG Nups, which shall be referred to as ‘like charge regions’ or LCRs. Our results on the distribution of FG motifs, polar residues, and LCR sequences demonstrate novel aspects of the formation of FG network at the center of the NPC. Our approach is verified by comparing the results against available data^5^,^18^,^19^,^39^.

## Results

### FG Nups do not share the same properties with other disordered proteins

**Figure 1:**
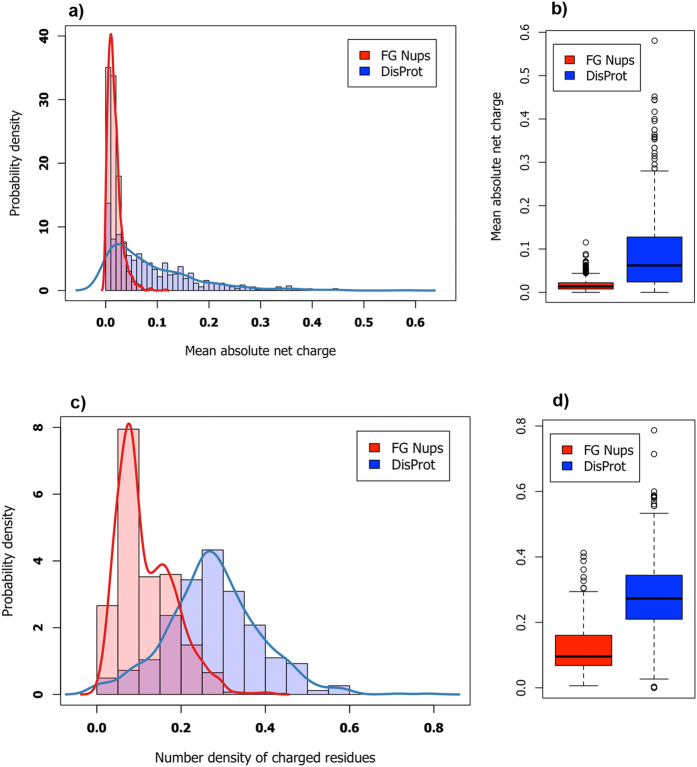
Comparison of FG Nups and DisProt proteins in terms of their charge content and distribution. Top Row: Probability density distribution (**a**) and box plot (**b**) of mean absolute net charge, defined as absolute net charge divided by the length of the sequence. FG Nups exhibit lower absolute net charge compared to the DisProt database–please refer to “Methods” for the definition of database of FG Nups and Disprot. Interestingly, DisProt proteins show a much wider range of mean absolute net charge compared to FG Nups, implying that FG Nups share the same charge content (number density and distribution) characteristics across species. Bottom Row: Probability density distribution (**c**) and box plot (**d**) of number density of charged residues, defined as the number of charged residues divided by the length of the entire sequence. Sequences of FG Nups feature lower density of charged residues compared to DisProt. The two comparisons imply that charged residues are not only responsible for the disorderedness of FG Nups, but are also carefully distributed to give specific functionalities to FG Nups. Note that the area under each curve equals one, which explains why the values on vertical axes could exceed unity.

The combination of low mean hydrophobicity and relatively high net charge are suggested to be the prerequisites for the lack of structure in IDPs^31^,^36^. In order to explore these properties in FG Nups, they were compared against proteins of the DisProt database using the charge-hydrophobicity plot^36^ (results not shown)–please refer to “Methods” for the definition of database of FG Nups and Disprot. Although FG Nups feature average hydrophobicity patterns similar to those in DisProt proteins (less than %7 difference on average), they exhibit significantly lower absolute net charge. The mean absolute net charge, i.e. absolute net charge divided by length of the sequence, was compared in FG Nups versus that in DisProt proteins (see Fig. 1). Interestingly, DisProt proteins show a considerably wider range of mean absolute net charge in their sequences compared to FG Nups. Average mean absolute net charge of FG Nups is about one-fifth of that in DisProt proteins. Therefore, it could be deduced that lower net charge is a shared feature of FG Nups across different species.

Most of the charged amino acids within the sequences of FG Nups are located within the FG linkers, i.e. domains that link FG motifs together. Analysis of these linker regions in FG Nups reveals that about 93% of them possess a net charge of either zero or one (Figure S1^14^).

Therefore, although net charge is suggested as a key parameter for distinguishing structured and disordered proteins^36^, in the case of FG Nups the total content (number density) of charged residues would be a more meaningful parameter^5^. Comparing number density of charged residues, i.e. number of charged residues divided by length of the sequence, between FG Nups and DisProt proteins demonstrates that average charge density of FG Nups is approximately one-half of that of DisProt proteins (see Fig. 1). Therefore, FG Nups not only have lower net charges but also exhibit significantly lower number density of charged residues as compared to DisProt proteins.

**Figure 2:**
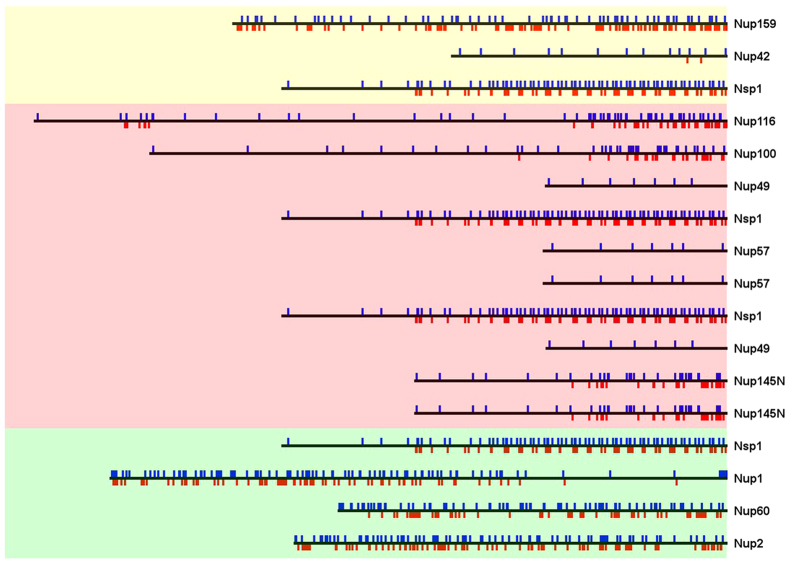
Distribution of charged residues along the sequences of yeast FG Nups. Positive charges are shown in blue while negative charges are shown in red. The far right side of the figure represents the NPC wall. FG Nups are drawn in their extended form. It is clearly observable that not all domains of FG Nups are rich in charged residues, which implicitly proposes specific functionalities for different regions of each FG Nup (also discussed in^5^ and^39^). Yeast is chosen as the example to represent the distribution of charged residues in the sequence of FG Nups since it is the most well studied species.

These findings are not completely in line with what is previously stated about FG Nups^42^. Although high net/total charge leads to an increase in the electrostatic repulsion within Nups^36^, it seems that, in the context of the NPC, charged residues are not solely responsible for disorderedness of FG Nups. To gain a better insight on the distribution of charges inside the nuclear pore, the distribution of positive and negative charges along the sequences of FG Nups in yeast NPC were examined (see Fig. 2). Evidently not all regions of FG Nups are rich in charged residues; rather there are regions that include very few charged residues. Consequently, one could speculate that disordered behavior is desired to the extent that it satisfies the function of an FG Nup. In line with this hypothesis, some FG Nups are observed to form local compact collapsed coil configurations to regulate nucleocytoplasmic transport^5^,^18^,^43^. For instance, Nsp1, one of the most studied FG Nups from yeast, possesses two distinct regions with one rich in FG motifs, i.e. FG domain, and the other rich in charged residues, i.e. stalk domain^5^,^18^. As claimed by Ando *et al.*^18^, stalk domains are responsible to extend all the way to the central axis of the pore, locating FG domains in the center of the pore to form the central transporter^5^,^18^. Central transporter is one of the proposed NPC transport mechanisms in which a network of FG Nups is formed in the center of the NPC. Active transportation needs the cargo complex to interact with this hydrophobic network to be able to pass through the nuclear pore^5^,^18^.

**Figure 3:**
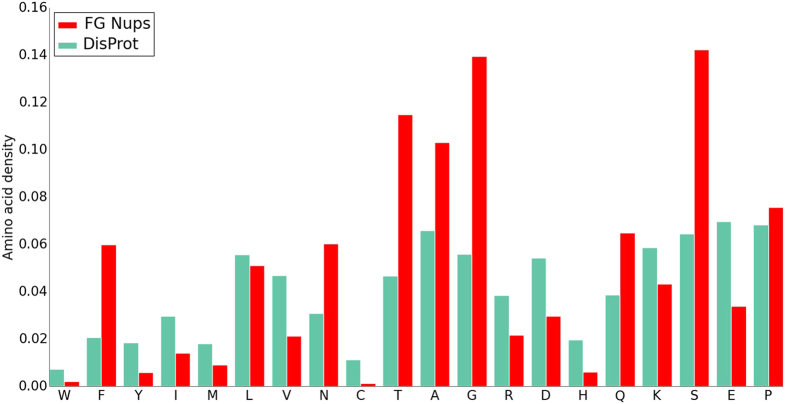
Amino acid abundance of FG Nups and DisProt proteins. Amino acids are sorted according to their disorder-promoting property, with Tryptophan as the most order-promoting amino acid and Proline as the most disorder-promoting one^44^. Both groups show low abundance of hydrophobic residues (Ile, Leu, Val, Trp, Tyr, Phe) compared to structured proteins. However, FG Nups have a lower content (number density) of hydrophobic residues compared to those in DisProt proteins, except for Phenylalanine. Furthermore, FG Nups demonstrate higher abundance of polar residues and lower abundance of charged residues compared to DisProt proteins. Abundance is defined as the number of residue normalized by the number of residues in the entire database.

**Figure 4:**
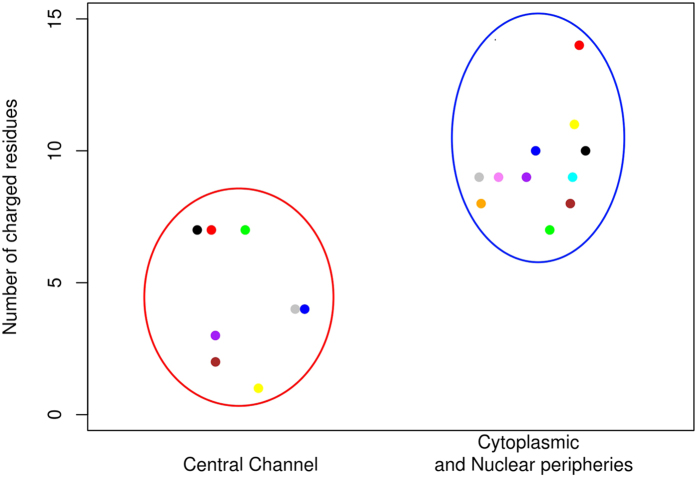
Comparison of central channel and periphery FG Nups in terms of number of charged residues embedded in LCR. Data points are jittered to enable the reader to observe all of the data points. FG Nups fall into two distinct groups, with the central channel group possessing LCRs of lower than eight and the periphery group containing LCRs of larger than seven residues. The analysis is conducted on FG Nups from yeast and vertebrates with known location, i.e. central channel or periphery. Analyzed FG Nups are: [Central Channel] Nsp1 (blue), Nup49 (black), Nup57 (red), Nup145N (green), Nup62 (purple), Nup54 (yellow), Nup45 (gray), Nup58 (brown), [Cytoplasmic and Nuclear peripheries] Nup1 (orange), Nup2 (violet), Nup159 (cyan), Nup42 (blue), Nup100 (black), Nup116 (red), Nup98 (green), Nup358 (purple), Nup214 (yellow), Nup153 (gray), Nup50 (brown). Location information is obtained from^52^.

Further analysis of amino acid abundance, defined as the number of residues in a certain group normalized by the number of residues in the entire database, was performed using FG Nups and DisProt databases and revealed more significant differences between the two groups. Amino acid abundance of the two groups sorted by disorder-promoting property of each amino acid with Tryptophan as the most order-promoting amino acid and Proline as the most disorder-promoting one^44^ is shown in Fig. 3. FG Nups are no exception to the fact that IDPs have a low content of hydrophobic residues (Ile, Leu, Val, Trp, Tyr, Phe) compared to structured proteins^45^,^46^. However, FG Nups show a significantly lower content (or number density) of hydrophobic residues as compared Fig. 4 to DisProt proteins except for Phenylalanine, since FG Nups are characterized by abundance of Phenylalanine and Glycine residues. One could speculate that FG Nups compensate for the order-promoting property of large number of Phenylalanine by lowering the number of other order-promoting residues. Among order-promoting amino acids, only Phenylalanine and Asparagine have higher abundance in FG Nups. Asparagine is a polar residue and interestingly FG Nups exhibit higher contents (or number densities) of all polar residues (Asn, Ser, Thr, Gln), likely to regulate FG linker interactions forming a network of FG Nups^14^. Furthermore, in line with the aforementioned comparison of charge content between FG Nups and DisProt proteins, the number density of individual charged residues is also lower in FG Nups compared to that in DisProt database.

Based on this sequence analysis of FG Nups, it could be concluded that in addition to FG motifs, charged and polar residues might be other important role-players in the behavior of FG Nups inside the pore. Therefore, a more in-depth analysis is conducted to extract unique patterns in the distribution of these types of residues along the sequence of FG Nups across all species.

### Like charge regions can partly explain characteristics of FG Nups network

Like charge regions (LCRs), also referred to as uncompensated charged groups^36^, are specific domains of protein sequences that contain only one type of charged residues, i.e. either positively or negatively charged. These regions are suggested to enhance the disorder behavior of IDPs^36^. We focus our bioinformatics analysis on largest LCRs, as they exhibit interesting evolutionary conserved characteristics (explained in the following sections). Therefore, to avoid confusion and for the sake of simplicity, hereafter, LCR refers to the largest LCR. The number of charged residues embedded in each LCR was studied among all FG Nups. The histogram of LCRs among all FG Nups is shown in Figure S2. FG Nups indicate a wide range of number of charges in LCRs mostly falling between two to ten charged residues. Investigating FG Nups with known locations, i.e. central channel or cytoplasmic and nuclear peripheries, reveals that FG Nups can be categorized into two groups; those located in peripheries possess LCRs of equal or more than seven charged residues, while this parameter is less than eight for central channel FG Nups (Figure 4). Since the location information of most of the FG Nups is lacking, this inference is based on a partial fraction of FG Nups (i.e. only those with experimentally verified localization data inside the NPC), however it could potentially offer a distinguishing parameter that helps isolate the two groups of FG Nups, i.e. central channel versus peripheral FG Nups. Further computational and experimental studies are indeed necessary to unravel the functional significance of number of charges in LCR.

**Figure 5:**
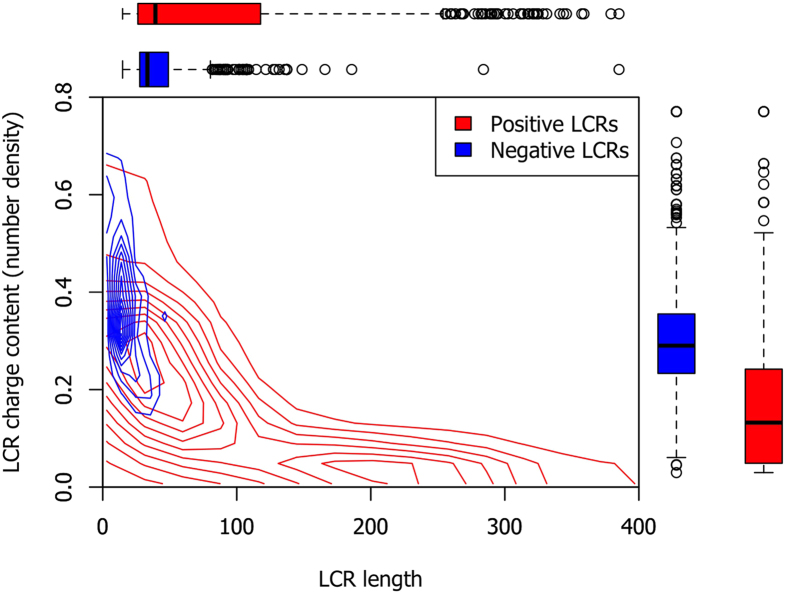
Distribution of FG Nups in length-content space of LCRs. Positive LCRs are shown in red while negative LCRs are shown in blue. Charge content here refers to the charge number density defined as number of charged residue normalized by the length of LCR. Negative LCRs are short and contain high charge density, while positive LCRs show very low charge density and longer sequences. In addition, positive LCRs have a wide range of lengths across different species.

Another interesting characteristic of LCRs is reflected in the distinction between positive and negative LCRs. The distribution of FG Nups within the length-content space of LCRs is shown in Fig. 5, where the horizontal axis is showing the length of LCRs and the vertical axis demonstrates the charge content (calculated as number density, i.e. number of charged residues per the length of the LCR) in that region. The length of each LCR is defined as the number of residues between the first and the last charged residue within the LCR. Interestingly, negative LCRs are distributed closely with short lengths and high content (number density) of charged residues. On the other hand, positive LCRs exhibit significantly lower content (number density) of charged residues. Furthermore, the positive LCRs are typically longer than the negative LCRs but show a wide range of lengths among different species. It could also be observed for yeast FG Nups in Fig. 2, with seven out of ten FG Nups including a long and low-content (low-density) positive LCR. Therefore, it can be concluded that this unique sequential feature of FG Nups is an evolutionarily conserved characteristic shared across all species. It is worth mentioning that clustering analysis reveals that positive LCRs have almost zero overlap with charged clusters (results not shown), emphasizing the low content (i.e. low number density) of positive LCRs in charged residues. This observation is in line with the results of a previous work on yeast FG Nups^5^. Dividing regions of FG Nups into low and high content charge domains, it was discovered that some yeast FG Nups only include high density charge regions, while some only include low density charge region, and some, e.g. Nsp1 and Nup1, include both high and low density charge regions^5^. Therefore, positive LCRs that have virtually no overlap with charge clusters correspond to low density charge regions, while charge clusters correspond to high density charge regions.

### Polar and charged residue contents are strongly correlated in FG linkers

**Figure 6:**
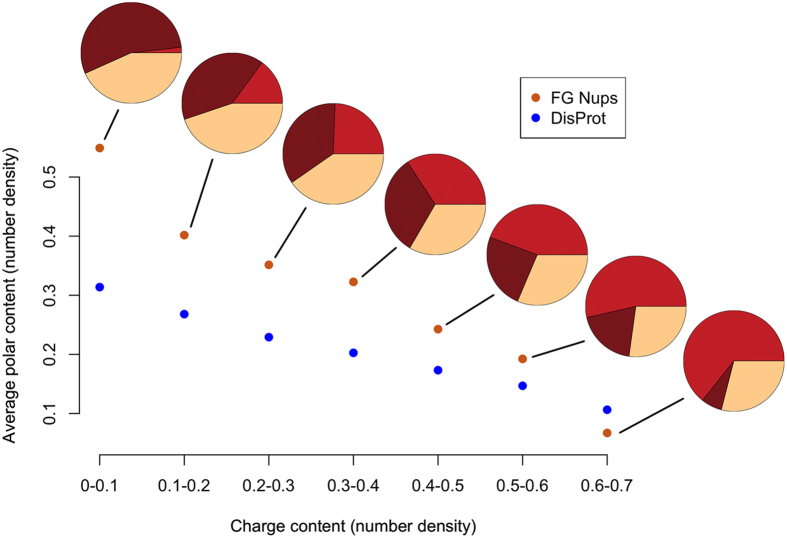
Average polar residue content versus charged residue content in FG Nups and Disprot proteins. For FG Nups, only FG linkers with the length of 10 to 30 residues are taken into account. For Disprot proteins, random sequences of 10–30 residues long are selected from Disprot proteins. For the sake of simplicity, these sequences from both groups are called linkers. Each point indicates the average number of polar residues among all linkers falling within that specific range of charged residue densities. Data points with at least 50 samples are shown (FG linkers with charged residue number densities of higher than 0.7 are excluded). Pie charts show average charged residue number density (red), average polar number density (dark brown), and average hydrophobic residue number density (cream) for the corresponding data point. Average number density of polar residues in FG Nups displays a sharp decreasing trend with respect to an increase in the number density of charged residues as compared to Disprot proteins, implying that FG Nups are more likely to have polar residues as the number of charged residues decreases. Disprot proteins are compared to FG Nups as a control group to show the significance of the observed evolutionarily conserved trend.

The histogram for the polar residue content in linker regions of 10-30 residues in length across all FG Nups is shown in Supplementary Figure S3. A wide range of polar residue number densities is exhibited in linker regions, varying from zero to almost 100% with a normal distribution around 50%. However, a more in-depth analysis of the distribution of polar residues shows a strong correlation between the number density of charged residues and polar residues in FG linkers. The average density of polar versus charged residues is shown in Fig. 6. It is clear that the higher number densities of charged residues in FG linkers tightly corresponds to a lower number density of polar residues. In order to show the significance of the evolutionarily conserved trend observed here, the same analysis was performed on a control group. The control group, Disprot proteins, exhibited a similar pattern but the trend was sharper in FG Nups, implying that FG Nups are more likely to have polar residues in their FG linkers when the number of charged residues decreases.

This conserved feature of FG Nups could be directly related to the results observed in computational studies on Nsp1^14^,^18^. Linker regions of Nsp1 are observed to interact via polar interactions^14^. Furthermore, the stalk domain of Nsp1, which is identified by linkers dense in charged residues with low densities of polar amino acids, forms a relaxed-coil configuration^18^. These observations collectively suggest that highly charged FG linkers with low densities of polar residues form relaxed coil configurations. On the other hand, FG linkers with a high density of polar residues and a low number density of charged residues act as facilitators for efficient interaction of FG motifs and form collapsed domains^14^,^18^.

### Clustering analysis reveals relative localization of FG motifs, charged and polar residues

**Figure 7:**
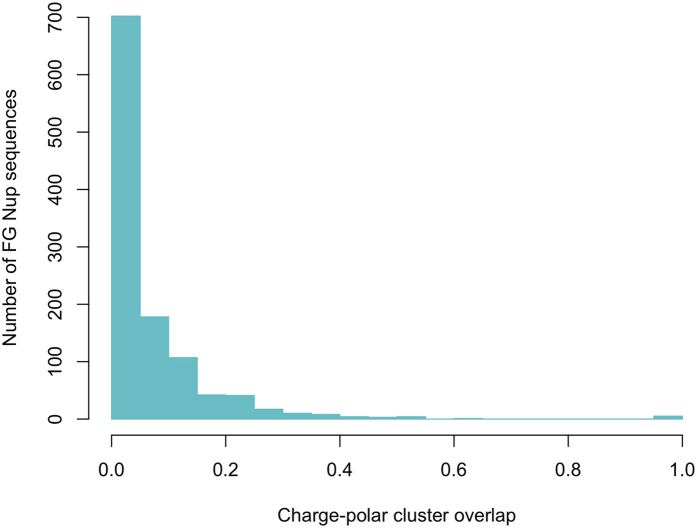
Overlap between charged and polar clusters. The relatively low overlap indicates that charge-rich regions, termed stalk domains^18^, have a low number density of polar residues. The overlap percentage calculation method is explained in the “Methods”, clustering method section. The same analysis was repeated on Disprot proteins as control group (Supplementary Figure S4). Relatively high overlap of polar and charged residues in Disprot shows the significance of the trend observed here.

**Figure 8:**
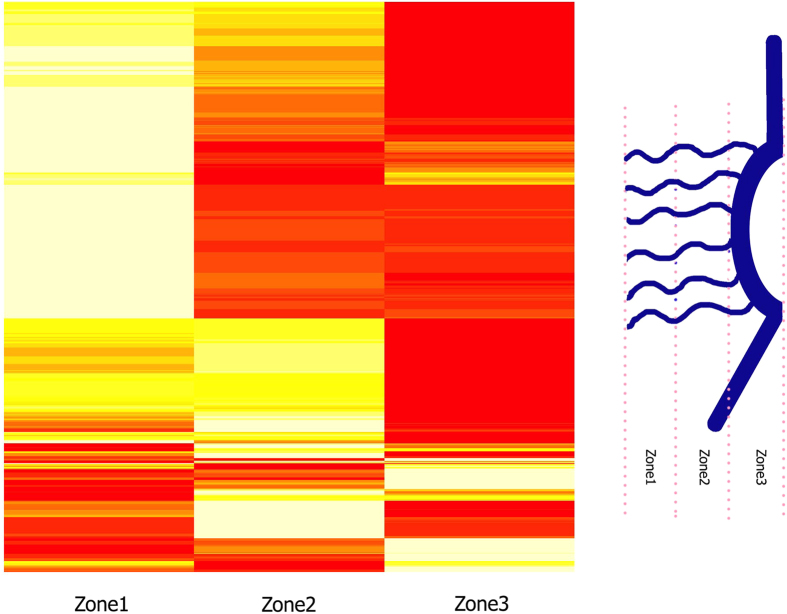
Distribution of polar clusters along the sequences of FG Nups. Right) A schematic showing half of the NPC from side-view. FG Nups are divided into three zones with zone1 representing the N-terminus and zone3 representing the C-terminus, which is mostly the structured region of the pore. Left). A heatmap showing abundance of polar clusters in the corresponding region. Brighter areas show higher content of polar clusters. FG Nups can be categorized into three groups. FG Nups of the first group are very rich in polar clusters in their first zone, while FG Nups of the second and third group have more polar clusters in their second and third zones, respectively.

**Figure 9:**
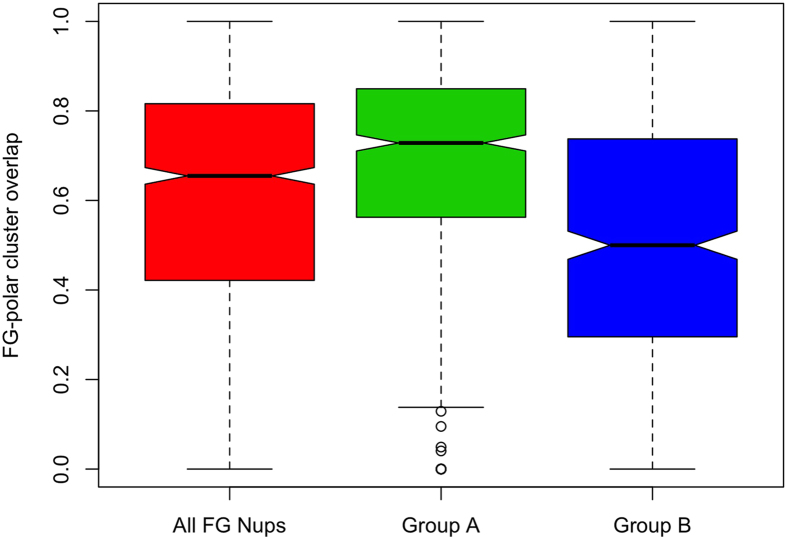
Overlap between FG and polar clusters in three different categories of FG Nups. Boxplots of the overlap among all FG Nups (red), FG Nups with more than half of their polar clusters located within the first zone (green), termed Group A, and FG Nups with more than half of their polar clusters located within second and third zones (blue), termed Group B are shown. Higher overlap of FG and polar clusters in group A implies a functional significance of overlapped FG-polar clusters at the tip of FG Nups.

A comprehensive study by Ando *et al.* 39 on FG clusters and charged clusters demonstrated that in 80% of the cases, there is no overlap between these clusters. In other words, they have a polarity in their location along the sequences of FG Nups. Here, we extend the analysis by taking into account the polar residues as well. Overlap analysis between clusters of charged and polar residues (Fig. 7) demonstrates a significant polarity between the two types of clusters. The low overlap indicates that charge-rich regions, named stalk domains^39^, are low in polar residue content. The same analysis was performed on a control group (Disprot proteins) and the results are shown in Supplementary Figure S4. The relatively high overlap between charged and polar clusters of Disprot proteins shows the significance of the evolutionarily conserved trend observed in Fig. 7. On the other hand, a similar analysis between clusters of FG motifs and polar residues (Supplementary Figure S5) demonstrates a wide range of overlap between FG and polar clusters. However, further analysis reveals a deeper relation between polar and FG clusters. To gain a better insight on the distribution of polar residues along FG Nups, a spatial analysis was performed on polar clusters by dividing FG Nup sequences into three zones (see Fig. 8), with zone1 representing the N-terminal tip near the central axis of NPC and zone3 representing the C-terminal end of FG Nups mostly embedded inside the NPC scaffold. The distribution of polar clusters along the sequences of FG Nups, is presented in Fig. 8 with brighter areas representing a higher number density of polar clusters. FG Nups are rarely rich in polar clusters in their third zone, implying that most FG Nups have a low density of polar residues in their C-terminus zone, which is a mostly structured region. On the other hand, most of the polar clusters are distributed in zones 1 and 2 with more FG Nups being rich in their first zone. While 56% of FG Nups have more than 50% of their polar clusters located in zone1, this value is only 19% for zone2. Based on this observation, we divided FG Nups into two groups according to abundance of polar clusters in the first zone. The FG Nups with more than 50% of their polar clusters located in zone1 are denoted as group A and the rest of the FG Nups as group B. Performing the same overlap analysis between FG and polar clusters of groups A and B (Figures S6 and S7), it is observed that FG Nups in group A have larger overlaps between FG and polar clusters. Group A shows a significantly higher overlap between FG and polar clusters (see Fig. 9). This observation implies a functional significance of overlapped clusters of FG and polar residues at the tip of FG Nups.

## Discussion

The intrinsic bimodality of sequences of some FG Nups has been previously proposed in several studies^5^,^18^,^39^. It was observed that FG and charged clusters are well separated within the sequences of FG Nups, forming two distinct regions with different physical behavior and function. However, previous studies were primarily based on charged residues and FG motifs, with some studies focusing only on a very few FG Nups. Here, we considered more than 1000 sequences of FG Nups and extended this hypothesis by extracting other specific features of these two regions along the sequences of FG Nups. We observed that charged clusters and polar clusters have little overlap. We also found specific low-density sequences of charged residues, named LCRs. In line with polar-charge cluster polarity, it was observed that positive LCRs have almost zero overlap with charged clusters. Collectively, one could therefore deduce that sequences of FG Nups are generally partitioned into two domains, one rich in charged residues and one including positive LCRs and a large fraction of polar residues along with FG motifs. This hypothesis is further supported by the overlap analysis between polar and FG clusters. It was previously observed that FG motifs are mainly clustered at the N-terminal tip of FG Nups^39^. We observed that polar clusters have a fair amount of overlap with FG clusters, however, FG Nups that have more than half of their polar clusters located within the first one-third of their sequence show higher overlap of FG and polar clusters. This observation implies that the presence of polar residues among FG motifs is more significant as we move toward the N-terminal tip of FG Nups, i.e. the central axis of NPC.

As mentioned, based on recent observations^5^,^18^,^39^, sequences of some of the FG Nups can be divided into two functional domains; stalk domain, which is highly charged and relatively straight, and an FG domain that possesses a compact structure and forms the central network of FG Nups, also known as the central transporter^5^. The stalk domain is responsible for bringing the FG domain near the central axis of the pore that is suggested to act as a hydrophobic core, facilitating transportation of cargo complexes via hydrophobic interactions^5^. Our results add to this model by proposing positive LCRs and polar residues as two major regulators of the central transporter. Since nuclear transport signals (NTRs) are negatively charged^25^, the presence of positively charged LCRs facilitates translocation of cargo complexes. It was also previously argued that electrostatic interactions are essential for selective transport^25^. In addition, electrostatic repulsion between like charges of LCRs can be considered as a regulatory mechanism that keeps the central network of FG Nups in a certain size. According to the forest model^5^, which suggests trafficking zones through the NPC, size of this network will significantly affect nucleocytoplasmic transport. Charged residues embedded in positive LCRs are sparse (Fig. 5) to prevent FG Nups from forming a relaxed coil structure while enabling them to maintain a certain radius of the central transporter. Furthermore, it can be postulated that LCRs allow the central transporter to possess sufficient amount of mobility and dynamic behavior via electrostatic repulsion of like charges, hindering hydrophobic forces from turning the network into a more compact structure. On the other hand, the presence of polar residues would improve the formation of the central network of FG motifs via polar interactions and facilitate cargo transport. This is supported by the computational study conducted on a number of segments of FG repeat sequences. It was observed that presence of hydrophilic linkers and polar residues promote FG repeat meshwork formation^14^.

**Figure 10:**
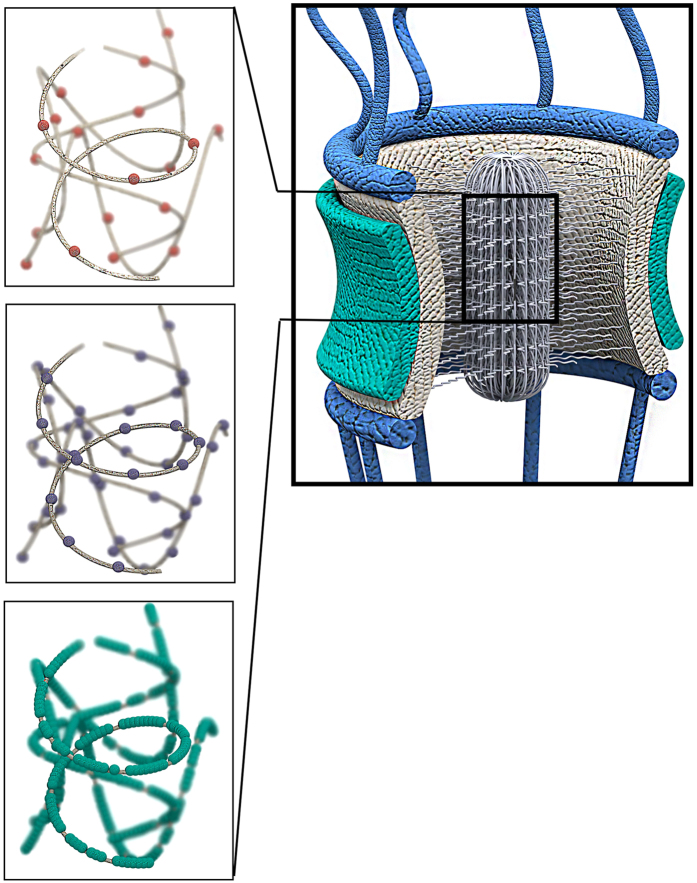
Schematic demonstrating cooperation of positive LCRs, FG motifs, and polar residues to form the FG network. Red spheres (top left box) represent positive charges embedded in positive LCRs, purple spheres (middle left box) represent FG motifs, and green spheres (bottom left box) represent polar residues within the central transporter. The highlighted FG Nups domain in the three left boxes represent N-terminal domain of NSP1, i.e. spheres represent location of charged (red) and polar (green) residues and FG motifs (purple). Due to the presence of positive LCRs, the central transporter is positively charged, facilitating transport of negatively charged NTRs. The dynamics of the central network of FG motifs is also regulated via charge repulsion in LCRs. In addition, low charge content of positive LCRs assists these domains of FG Nups to form a collapsed coil conformation. Furthermore, high densities of polar residues among FG repeats promote the hydrophobic interactions and meshwork formation. On the other hand, highly charged stalk domains of FG Nups, which are depleted of polar residues, place the central transporter in the center of the NPC by forming a relaxed coil conformation.

Collectively, our bioinformatics study on sequences of FG Nups enables us to divulge the role of polar residues and LCRs in the formation of the central transporter, which was not previously examined. The sequential features discussed above are cooperating together to enhance nucleocytoplasmic transport. Cooperation of positive LCRs, FG motifs and polar residues is schematically summarized in Fig. 10. The central transporter is positively charged and is dynamic enough due to the charge repulsion in LCRs. In addition, low charge densities of positive LCRs assists these domains of FG Nups to form a collapsed coil conformation^5^. Furthermore, the central transporter has a high density of polar residues to promote FG repeat hydrophobic interaction and meshwork formation. Finally, the central FG network is placed at the center of the nuclear pore by the other domain of proteins that have a high number density of charged residues and a low density of polar residues. All these sequential features are extracted from more than a thousand FG Nups across 252 species and therefore the results can be considered as the evolutionarily conserved features and function of the NPC.

Although this study cannot predict the doughnut distribution of FG repeats previously seen in experimental studies and coarse-grained simulations^19^,^47^, our results are in general agreement with those studies. According to their observation, while charged residues are mostly located near the wall, FG repeats form a doughnut around the central axis. This is in agreement with our results as well as previous studies^5^,^18^,^39^ that divide most of the FG Nups into two distinct domains with one richer in FG motifs and the other richer in charged residues. In fact, while our study suggests that FG repeats are placed at the center of the pore, it does not predict the shape they form at the center, whether a central plug or a doughnut. Furthermore, it was observed that replacement of charged residues with neutral ones results in a high density, wide distribution of all FG repeats inside the pore^19^. This is in line with the hypothesis that relaxed coil conformation of charge-rich domains places FG domains at the center of the NPC. In addition, random shuffling of FG Nup sequences completely disturbs formation of the dense FG region^19^, which is consistent with our results that the specific distribution of different types of residues is the key parameter in FG network formation.

Interestingly, a small fraction of LCRs are negatively charged, with a short length and high number density of charged residues (Fig. 5). In yeast, specifically, LCRs of all FG Nups are positive, except for the three outermost FG Nups, namely Nup159, Nup 1 and Nup2. Therefore, it could be postulated that these FG Nups, when compared to central channel FG Nups, play a distinct role in nucleocytoplasmic transport. Being located at the outermost regions of the pore, these FG Nups are primarily responsible for attracting or rejecting importins and exportins to initiate nucleocytoplasmic transport. As a result, it is not desirable for them to form collapsed coil configurations. Moreover, it can be speculated that the straightened structure of these FG Nups enables them to explore a larger space and attract cargos more easily and deliver them to the central channel where the central transporter has been formed. It is important to note that, as the length of these negative LCRs are short, and the rest of the sequence is full of both positive and negative charges, the electrostatic repulsion between the negative LCRs and negative NTRs should not have a significant negative effect on binding of NTRs and FG Nups.

Our bioinformatics study was conducted on all FG Nups across all different species. It remains an open question to explore how the presence of multiple cargo complexes inside the nuclear pore and their cross-interactions with FG Nups can potentially influence the conformational dynamics of the FG Nups. This calls for further computational and experimental studies to unravel the underlying mechanisms.

## Methods

Most analyses presented herein were conducted via in-house Python scripts, except disorder prediction and clustering, which were conducted through ESPRITZ Web server (version 1.3) and ELKI data mining software, respectively^48^,^49^. Considering the high risk of generating deceiving results, studies were initiated by comprehensive validation and verification steps. All results were verified against previous studies^5^,^31^,^39^. Data visualization was performed using Python and R scripts.

### Protein datasets

Two groups of proteins were analyzed in this study; the first group, named FG Nups, was initially taken from a recent bioinformatics study^39^, including Nups from 252 species. The database was then updated according to the latest changes made to the Uniprot database until March 2014, eventually including 1138 FG Nups. The database was originally created by extracting entries associated with the keyword ‘nucleoporin’ from the Uniprot database, and selecting those with a high percentage of disorder (>30%) and high percentage of FG motifs (>0.15 FG/AA) as FG Nups^39^. All studies on FG Nups were conducted solely on their disordered regions, unless otherwise specified. Therefore, hereafter, FG Nups refer to disordered domains of these proteins. The second group, termed DisProt, included more than 1500 disordered regions of 694 proteins adapted from DisProt database^41^. DisProt is a database of proteins that contain at least one experimentally determined disordered domain along their sequence. Disordered domains were extracted from the latest version of the database, i.e. version 6.02.

### Sequence analysis

Mean absolute net charge was defined as absolute net charge along the sequence of a protein divided by its length, with residues “R” and “K” as positive charges and residues “D” and “E” as negative charges. All charged residues were assumed to have the same amount of charge. Mean hydrophobicity was defined as total hydrophobicity of the sequence of the protein divided by its length. Hydrophobicity scale proposed by Kyte and Doolittle^50^ was used, with hydrophobicity of each individual amino acid normalized to a scale of 0 to 1^31^. FG linkers in FG Nups were characterized by being located between two FG motifs and consisting of 10 to 30 residues. In Fig. 6, in order to show the significance of the observed trend, the results are compared to those of Disprot proteins. Random sequences of 10–30 residues long were chosen to be compared to FG linkers from FG Nups. Like charge regions (LCRs) were determined by multiple charged residues of the same sign that are not interrupted with charged residues of the opposite sign.

### Clustering analysis

For clustering purposes, initially, motifs/residues of interest, i.e. FG motifs, charged residues, and polar residues were located along the sequence of FG Nups. FG clusters were identified by “FG” and “GF” motifs while charged and polar clusters were determined by “D, E, R and K” and “S, N, T, and Q”, respectively. Clustering was conducted with a density based method. PreDeCon clustering algorithm was used through ELKI data mining software^49^. Clustering parameters of this algorithm, i.e. minimum points and epsilon, were tuned to maximize Dunn’s validity index^51^ and they were found to be 35, 8, and 2 for FG Nups, charged residues, and polar residues, respectively. Minimum points were set to two, one motif/residue for each boundary with the exception of polar residues, due to their compact distribution, with four minimum points. We found that on average, FG, charged, and polar clusters contain 128, 25, and 5 amino acids, respectively. The size of charged and FG clusters are consistent with the results reported by^39^. The sizes of the clusters are reversely correlated with the content (number density) of that residue type in the sequence. As polar clusters are very abundant in FG Nups, the average size of the cluster is very small relative to charged residues and FG motifs that are less abundant.

Overlap between a specific type of cluster with other clusters was quantified by the total number of residues shared between them divided by the sum of the sizes of the desired cluster type in each FG Nup. Residue location approximation was performed on whole sequences of FG Nups. The sequence of each FG Nup was divided into three zones, with the first zone located on the N-terminus and the third one on the C-terminus. Therefore, zone3 approximately represents the structured region of the sequence embedded in the scaffold, while zones two and one represent near the wall and near the central axis regions of FG Nups inside the NPC, respectively. For each zone, the proportion of the clusters contained within that zone was calculated. For instance, if 10 residues of one cluster were in the first zone, and there were 50 residues in that cluster type in the sequence of FG Nup, the proportion of that cluster type in the first zone one would be 10/50 = 0.2.

## Additional Information

**How to cite this article**: Peyro, M. *et al.* Evolutionarily Conserved Sequence Features Regulate the Formation of the FG Network at the Center of the Nuclear Pore Complex. *Sci. Rep.* **5**, 15795; doi: 10.1038/srep15795 (2015).

## Supplementary Material

Supplementary Information
