# Supplementary material for: Evolutionarily Conserved Sequence Features Regulate the Formation of the FG Network at the Center of the Nuclear Pore Complex
Source: Sci Rep. 2015 Nov 6;5:15795. doi: 10.1038/srep15795 (PMC4635341; doi:10.1038/srep15795)
Supplement: Supplementary Information [file srep15795-s1.pdf]

Evolutionarily Conserved Sequence Features Regulate the Formation of FG Network at the Center of the Nuclear Pore Complex

(Supplementary Information)

M. Peyro[1], M. Soheilypour[1], B.L. Lee, M.R.K. Mofrad*

Department of Bioengineering, University of California, Berkeley, CA 94720

[1] These authors contributed to this paper equally.

*Corresponding Author
Mohammad Mofrad, PhD
Professor, Department of` Bioengineering
University of California Berkeley
208A Stanley Hall #1762
Berkeley, CA 94720-1762
Phone: (510) 643-8165
Fax: (510) 642-5835
Email: mofrad@berkeley.edu
http://biomechanics.berkeley.edu

Keywords: Nuclear Pore Complex, Nucleoporins, Bioinformatics, Sequence Composition, Polar Residues, Charged Residues

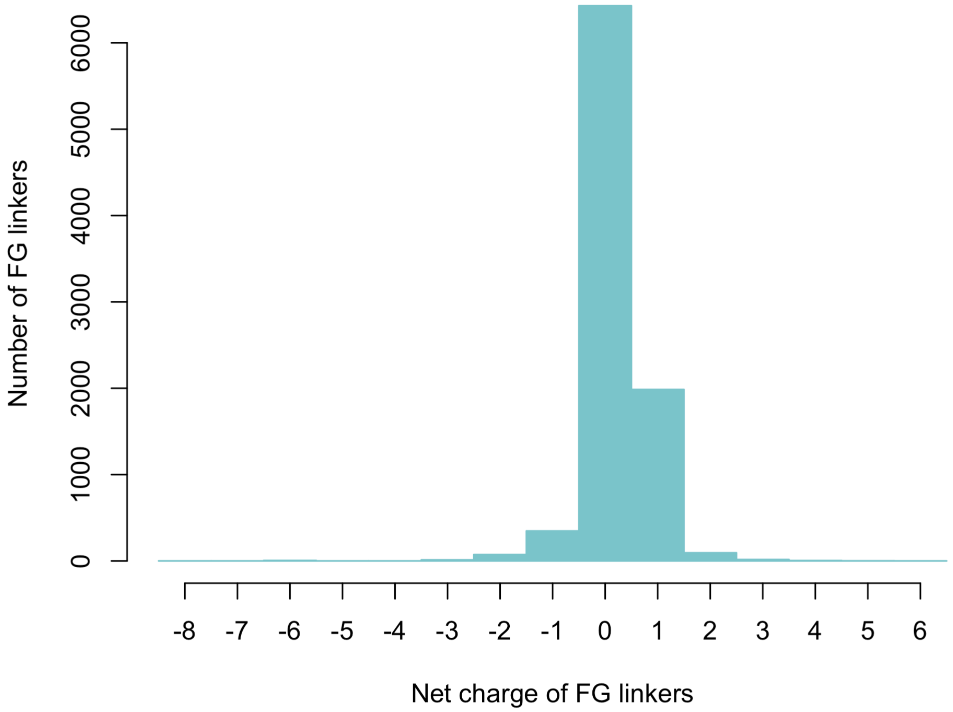

Figure S1: Histogram of net charge of FG linkers. More than 90% of FG linkers possess a net charge of either zero or one. Therefore, although net charge is considered as a primary parameter for distinguishing ordered and disordered proteins [1], in the case of FG Nups, the total number density of charged residues would be a more meaningful parameter [2].

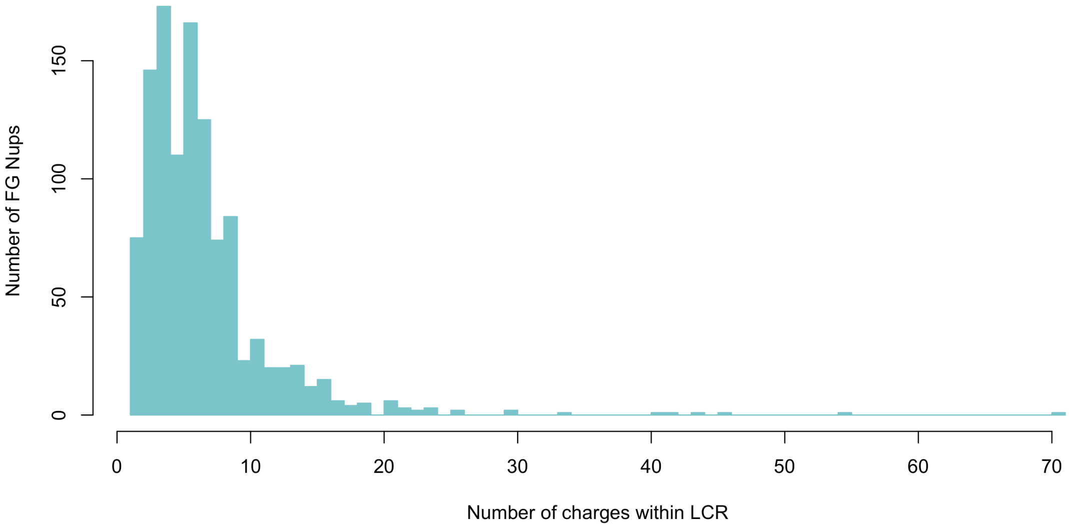

Figure S2: Histogram of number of charged residues within LCRs among all FG Nups. Although FG Nups indicate a wide range of number of charges in LCRs, location analysis of FG Nups with known location, i.e. central channel or cytoplasmic and nuclear peripheries, reveals that FG Nups can be grouped into two categories; those located in the peripheries possess LCRs of equal or more than seven charged residues, while this is less than eight for central channel FG Nups (see Figure 4).

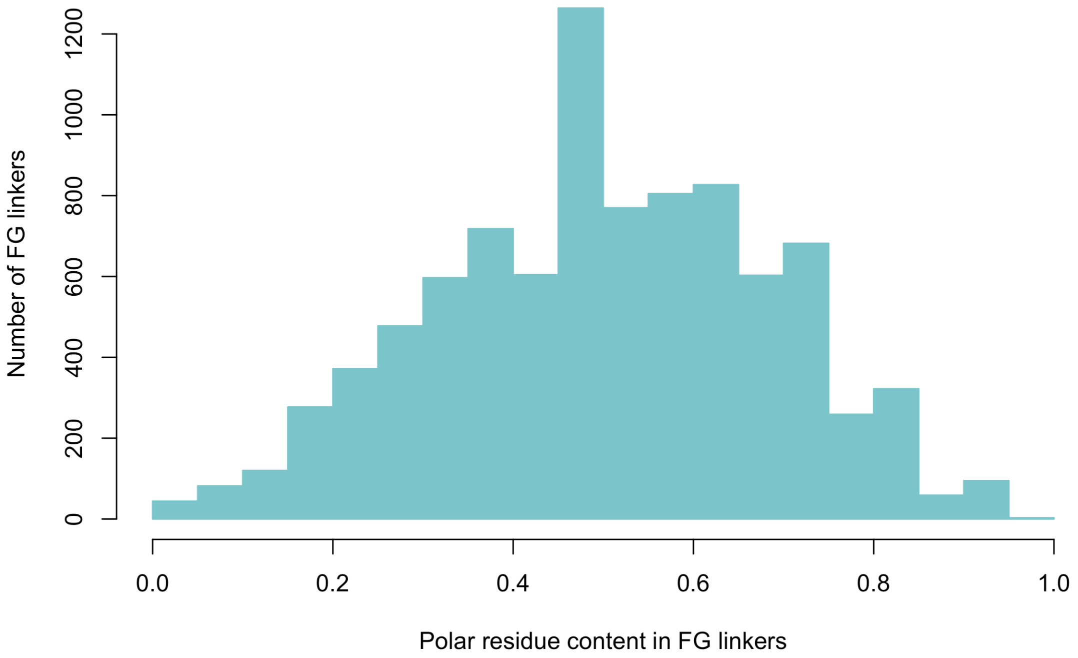

Figure S3: Histogram of polar residue content within FG linkers of 10-30 residues long. FG linkers exhibit a wide range of polar content with approximately a normal distribution about 50%. However, in-depth analysis of FG linkers reveals a strong relation between number density of charged and polar residues within the sequence of FG linkers.

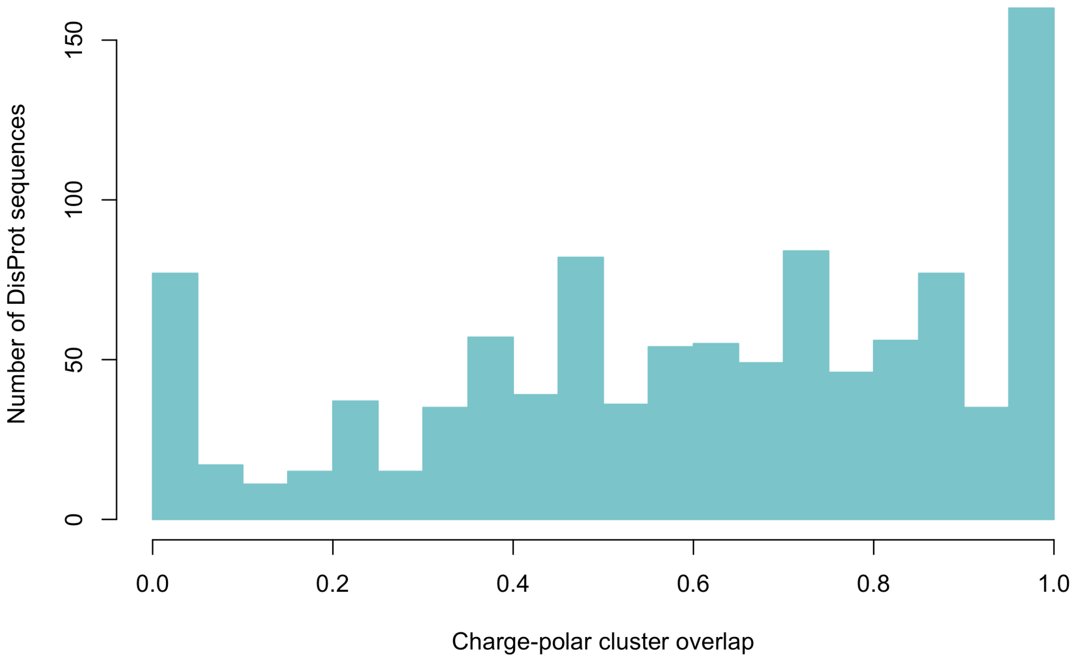

Figure S4: Overlap between charged and polar clusters of Disprot. The relatively high overlap for the control group shows the significance of the trend found in Fig. 7 for FG Nups.

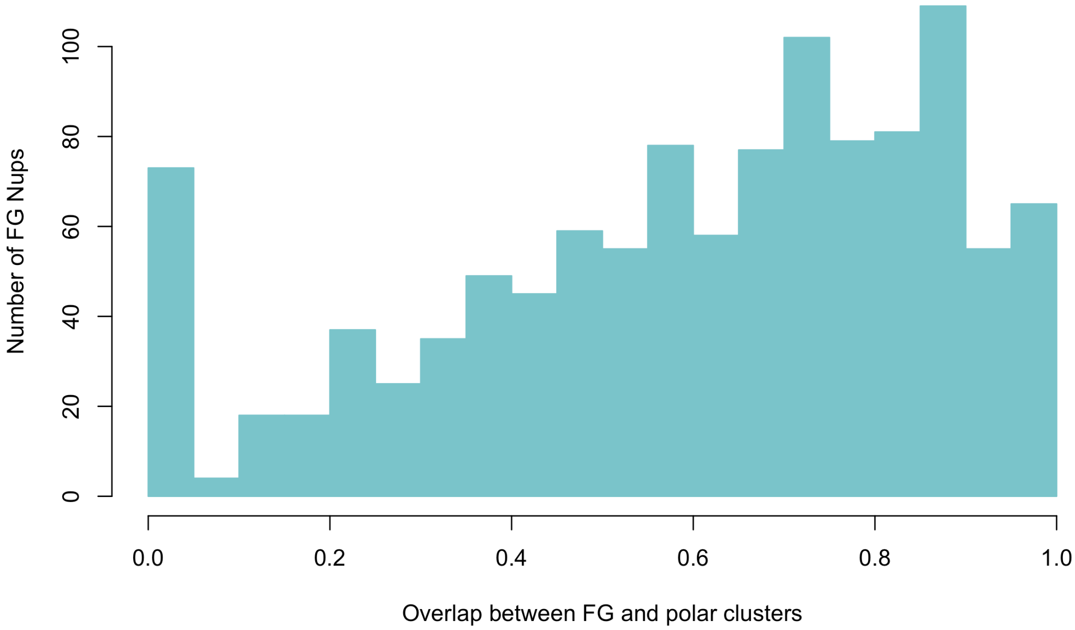

Figure S5: Overlap between FG and polar clusters. Although FG Nups show a wide range of overlap between FG and polar clusters, further analysis reveals that FG Nups with more than half of their polar clusters located in the first one-third of their sequence exhibit a higher overlap between FG motifs and polar residues, which implies a potential functional significance of overlapped FG-polar clusters at the tip of FG Nups.

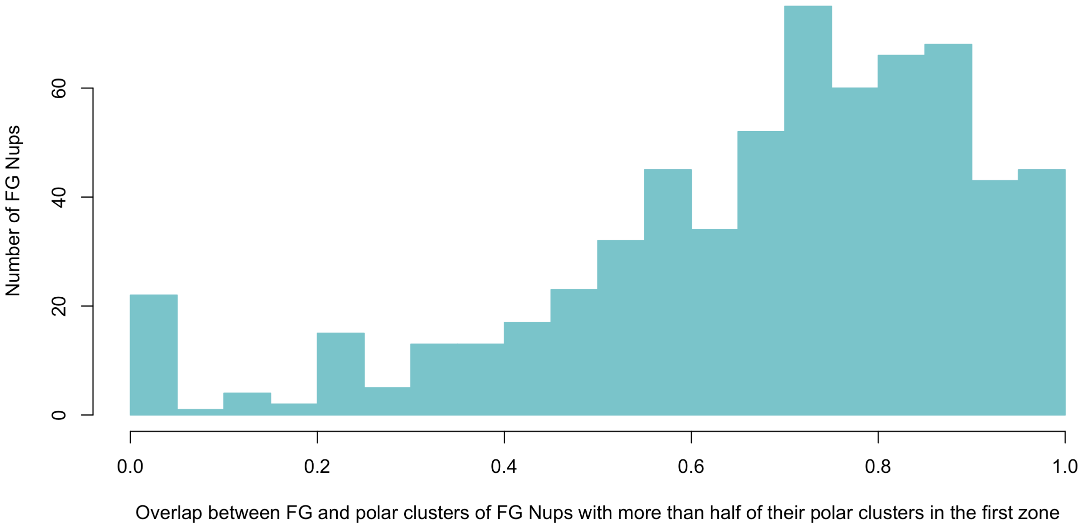

Figure S6: Overlap between FG and polar clusters of FG Nups with more than half of their polar clusters located in the first section of their sequence.

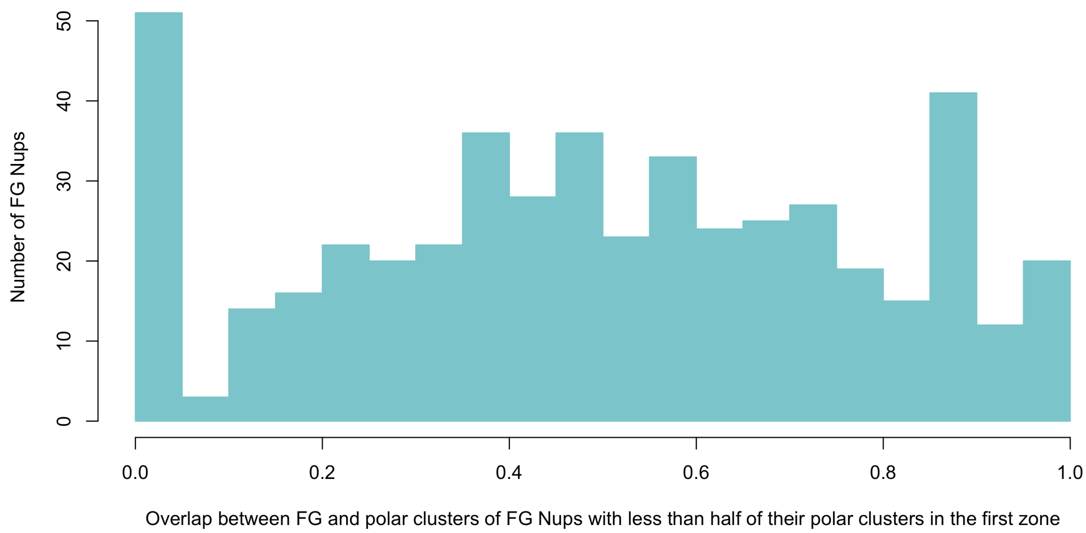

Figure S7: Overlap between FG and polar clusters of FG Nups with less than half of their polar clusters located in the first section of their sequence.

References

[1] Uversky, V. N. Unusual biophysics of intrinsically disordered proteins. *BBA-Proteins Proteom.* **1834(5),** 932-951 (2013).

[2] Yamada, J., et al. A bimodal distribution of two distinct categories of intrinsically disordered structures with separate functions in FG nucleoporins. *Mol. Cell. Proteom.* **9(10),** 2205-2224 (2010).
